# Genomics of lethal prostate cancer at diagnosis and castration resistance

**DOI:** 10.1172/JCI132031

**Published:** 2020-02-24

**Authors:** Joaquin Mateo, George Seed, Claudia Bertan, Pasquale Rescigno, David Dolling, Ines Figueiredo, Susana Miranda, Daniel Nava Rodrigues, Bora Gurel, Matthew Clarke, Mark Atkin, Rob Chandler, Carlo Messina, Semini Sumanasuriya, Diletta Bianchini, Maialen Barrero, Antonella Petermolo, Zafeiris Zafeiriou, Mariane Fontes, Raquel Perez-Lopez, Nina Tunariu, Ben Fulton, Robert Jones, Ursula McGovern, Christy Ralph, Mohini Varughese, Omi Parikh, Suneil Jain, Tony Elliott, Shahneen Sandhu, Nuria Porta, Emma Hall, Wei Yuan, Suzanne Carreira, Johann S. de Bono

**Affiliations:** 1 Vall d’Hebron Institute of Oncology (VHIO) and Vall d’Hebron University Hospital, Barcelona, Spain.; 2 The Institute of Cancer Research, London, United Kingdom.; 3 The Royal Marsden NHS Foundation Trust, London, United Kingdom.; 4 Instituto Oncoclinicas-Grupo Oncoclinicas, Rio de Janeiro, Brazil.; 5 The Beatson West of Scotland Cancer Centre, Glasgow, United Kingdom.; 6 University College Hospital, London, United Kingdom.; 7 St James’s University Hospital, Leeds, United Kingdom.; 8 Musgrove Park Hospital, Taunton, United Kingdom.; 9 Royal Blackburn Hospital, Blackburn, United Kingdom.; 10 Belfast City Hospital, Belfast, United Kingdom.; 11 The Christie Hospital, Manchester, United Kingdom.; 12 Peter McCallum Cancer Center, Melbourne, Australia.

**Keywords:** Cell Biology, Oncology, Cancer, Molecular biology, Prostate cancer

## Abstract

The genomics of primary prostate cancer differ from those of metastatic castration-resistant prostate cancer (mCRPC). We studied genomic aberrations in primary prostate cancer biopsies from patients who developed mCRPC, also studying matching, same-patient, diagnostic, and mCRPC biopsies following treatment. We profiled 470 treatment-naive prostate cancer diagnostic biopsies and, for 61 cases, mCRPC biopsies, using targeted and low-pass whole-genome sequencing (*n* = 52). Descriptive statistics were used to summarize mutation and copy number profile. Prevalence was compared using Fisher’s exact test. Survival correlations were studied using log-rank test. *TP53* (27%) and *PTEN* (12%) and DDR gene defects (*BRCA2* 7%; *CDK12* 5%; *ATM* 4%) were commonly detected. *TP53*, *BRCA2*, and *CDK12* mutations were markedly more common than described in the TCGA cohort. Patients with *RB1* loss in the primary tumor had a worse prognosis. Among 61 men with matched hormone-naive and mCRPC biopsies, differences were identified in *AR*, *TP53*, *RB1*, and PI3K/AKT mutational status between same-patient samples. In conclusion, the genomics of diagnostic prostatic biopsies acquired from men who develop mCRPC differ from those of the nonlethal primary prostatic cancers. RB1/TP53/AR aberrations are enriched in later stages, but the prevalence of DDR defects in diagnostic samples is similar to mCRPC.

## Introduction

Interpatient genomic heterogeneity in prostate cancer is well-recognized (1). However, molecular stratification of prostate cancer to guide treatment selection based on predictive genomic biomarkers remains an unmet clinical need. Recent genomic studies have elucidated this interpatient heterogeneity, identifying multiple potentially actionable alterations which are now being evaluated in clinical trials. These studies have also described differences in the genomic landscape of the different clinical states of the disease (localized vs metastatic) (1, 2). Alterations in the *AR* gene (mutations, amplifications, and structural variants) are more prevalent in metastatic castration-resistant prostate cancer (mCRPC), and are associated with the development of castration-resistance as well as resistance to abiraterone acetate and enzalutamide (3, 4). Moreover, loss-of-function events in *TP53*, *RB1*, *PTEN*, and DNA damage repair (DDR) genes are more common in mCRPC compared with nonmetastatic prostate cancer cohorts. It remains unclear whether these differences are the result of evolutionary processes in response to therapy exposure, or whether these reflect different disease subtypes with differing outcomes.

An ultimate aim of understanding the genomic landscape of cancer is the implementation of more precise therapeutic strategies, but metastatic biopsy acquisition is a key obstacle for implementing genomic stratification in clinical practice. Liquid biopsies can partially overcome this limitation, but these assays are not yet validated to replace tumor biopsy testing, at least for prostate cancer (5, 6). Understanding if primary tumor biopsies can be used for molecular stratification to guide the treatment of advanced mCRPC years later remains a key question.

This study aims to describe the genomic profile of primary tumor biopsies from lethal prostate cancers, either presenting as metastatic hormone treatment–naive prostate cancers, or locoregional tumors that later evolve to metastatic disease. We hypothesized that these primary tumors would be enriched for alterations previously associated with mCRPC and would be different to those primary prostate tumors that do not recur. Additionally, we assessed a cohort of same-patient, matched, treatment-naive, and mCRPC biopsies to determine if these genomic defects change during treatment with tumor evolution.

## Results

### Patient and sample disposition.

Between March 2015 and December 2017, 652 primary tumor samples from consenting patients were received; 87 cases (13%) were discarded due to either low DNA yield or excessive DNA degradation. Hence, targeted next-generation sequencing (NGS) was successfully performed on 565 prostate cancer diagnostic biopsies. Fifty-four cases were excluded due to either the biopsy not being collected before ADT, or diagnosis being based on a metastatic biopsy (Supplemental Figures 1 and 2 in the Supplemental Material; supplemental material available online with this article; https://doi.org/10.1172/JCI132031DS1). We analyzed the NGS of the remaining 511 samples; of those, 41 (8%) cases did not meet quality control criteria for copy-number calling (7) and were discarded, so the final analysis evaluated 470 cases. Two cohorts were defined for the planned analyses based on disease extent at the time of original diagnosis: cohort 1 was composed of 175 cases with locoregional prostate cancer at diagnosis (69.5% confined to the prostate, 30.5% with pelvic nodal extension); cohort 2 included 292 primary tumors from patients with metastatic disease at diagnosis. The clinical records of 3 subjects were unobtainable (Table 1).

### Genomic profile of lethal primary prostate tumors.

**Figure 1:**
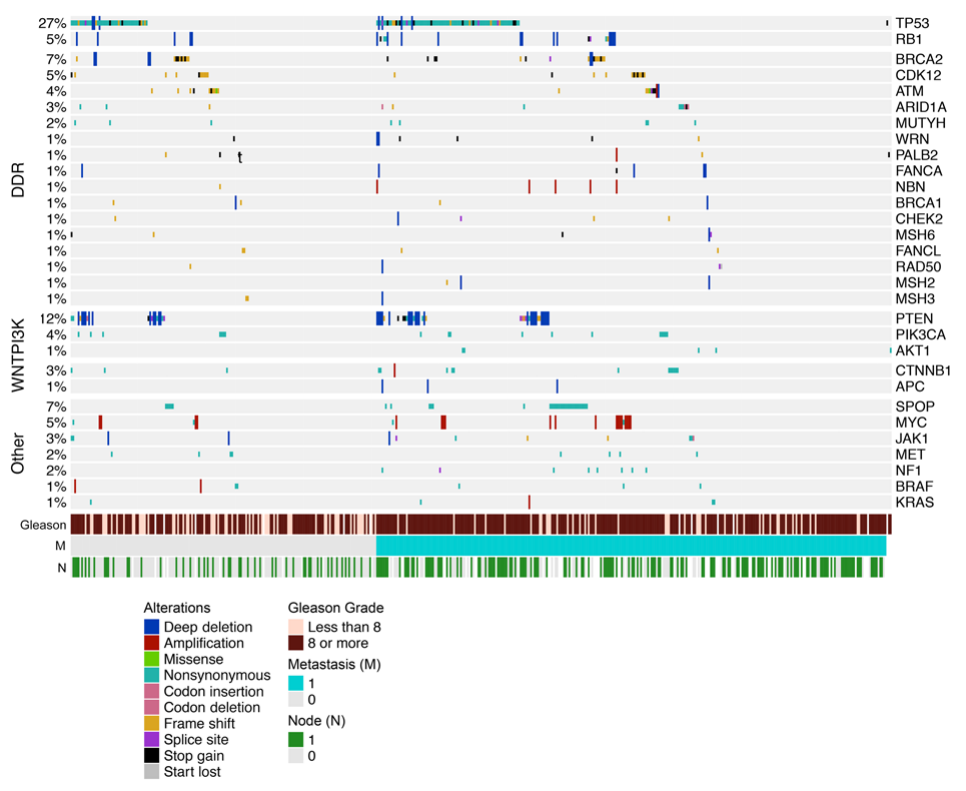
Oncoprint of genomic aberrations. The oncoprint includes nonsense, indels, splice site mutations, relevant missense mutations, and copy number changes for 470 untreated primary prostate cancer biopsies from patients who later developed metastatic castration-resistant disease.

Recurrent aberrations in genes and pathways related to lethal prostate cancer were identified, the commonest being mutations and homozygous loss of *TP53* (27%) (Figure 1 and Supplemental Figure 3). Deleterious mutations and/or homozygous deletions in genes involved in DDR pathways were identified in 23% of primary tumors. *BRCA2* was the DDR gene most commonly altered (7%). Alterations in mismatch repair genes were detected in 11 of 470 (2%) cases.

Activating mutations in *PIK3CA* and *AKT1* were detected in 5% of tumors, with *PTEN* loss-of-function mutations or deep deletions in 12% of tumors. Deep deletions of *RB1* were uncommon in the primary tumors (5%), although shallow deletions in *RB1* were frequent. Genes in the WNT pathway (loss of *APC* or activating mutations in *CTNNB1*) were altered in 7% of cases (8, 9). SPOP mutations were identified in 7% cases (10, 11). Surprisingly, low-allele frequency *AR* T878A or R630Q mutations (always with low mutation allele frequency, range 0.06–0.18) were detected in 1% of treatment-naive samples (12).

Our cohort 1 of primary tumors, without detectable metastases at diagnosis, was enriched for alterations in *TP53* (25% vs 8%; *P* < 0.001), *BRCA2* (8% vs 3%; *P* = 0.015), and *CDK12* (6% vs 2%; *P* = 0.04) when compared with the TCGA series (Table 2). Conversely, *SPOP* mutations were less common in our population than in the better prognosis TCGA series (3% vs 11%; *P* = 0.001). No relevant differences in prevalence of other mutations were observed when comparing cohort 1 and cohort 2. After adjusting for Gleason score, CDK12 mutations were enriched in Gleason 8 or higher cases (1 of 105 cases in Gleason 6–7 vs 21 of 353 in Gleason ≥8) (Supplemental Table 3).

### Clinical outcome based on primary tumor genomics.

Median time to ADT progression and start of first mCRPC therapy was 1.17 years (95% CI: 1.08–1.26 years) among the subset (*n* = 210) of patients with clinical data available. Median overall survival from first evidence of metastatic disease was 4.28 years (95% CI: 3.72–4.84 years).

None of the gene alterations were associated with a significantly different time to ADT progression; patients with germline or somatic *BRCA2* alterations had the lowest median time to ADT progression among the subgroups but the differences were not significant (median 0.92 years; 95% CI: 0.5–1.17; *P* = 0.39) (Table 3).

Patients with *RB1* alterations in the primary tumor had a significantly shorter overall survival (OS) (median OS from metastatic disease 2.32 years; 95% CI: 1.82–3.84; *P* = 0.006) (Table 3 and Supplemental Figure 4).

### Changes when assessing clinically actionable genomic alterations in patient-matched treatment-naive and castration-resistant samples.

We pursued NGS of mCRPC biopsies acquired from 61 patients participating in this study to further investigate if certain gene aberrations were detected more often in biopsies after progression on ADT and subsequent lines of therapy. Overall, we performed targeted NGS on 61 mCRPC biopsies (using the same panel as for the primary treatment-naive samples). Copy-number profiles for both primary and mCRPC samples were compared using low-pass whole-genome sequencing (WGS) in 52 cases with sufficient DNA in both samples. Copy number estimation was adjusted for ploidy and tumor purity, since mCRPC biopsies had higher tumor content overall than the primary prostate biopsies (Supplemental Figures 5 and 6).

The median time between the 2 same-patient biopsies was 45.2 months (range 12–211 months). All mCRPC samples were obtained after progression on ADT, and in 50 of 61 (82%) cases after progression on at least 2 further lines of therapy for mCRPC (80% after at least 1 taxane and 90% after abiraterone acetate and/or enzalutamide) (Table 4).

**Figure 2:**
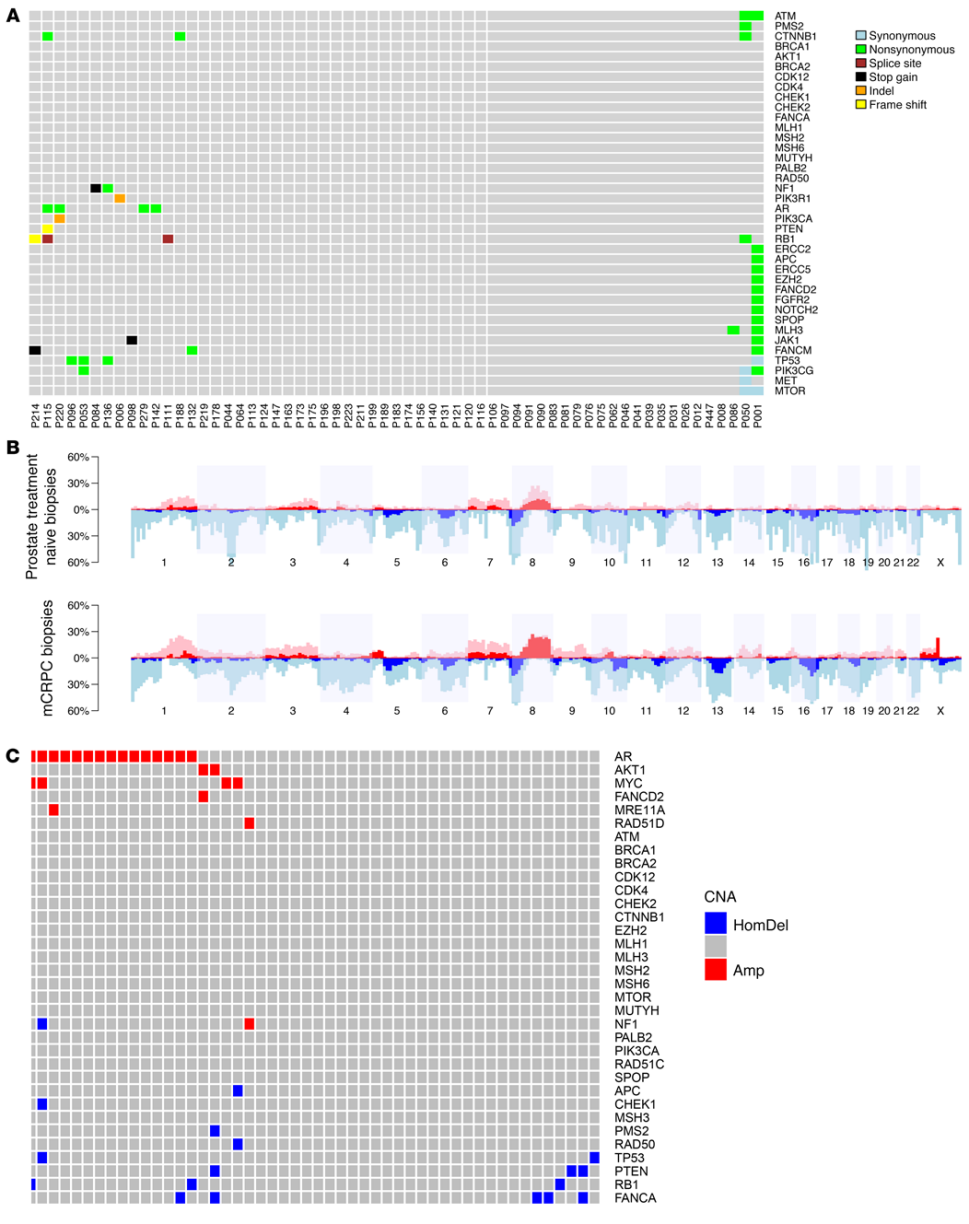
Differences in genomic profiles among same-patient, matched, primary-untreated, and mCRPC biopsies. (**A**) Mutation calls in genes of interest for the mCRPC biopsies which were not present in the treatment-naive primary tumor for the same patient (61 pairs, full gene set in Supplemental Figure 6). (**B**) Overall copy number profiles based on low-pass WGS (52 pairs). (**C**) Amplifications (Amp) and deep deletions (HomDel) detected in the mCRPC biopsies and not present in the treatment-naive primary tumors for the same patient (based on low-pass WGS, after adjusting for tumor purity and ploidy, and validated by SNP data from targeted panel sequencing).

The most common finding, when comparing same-patient primary treatment-naive and mCRPC samples, was an increase in AR mutations and amplification. Other than *AR*, the main differences between the 2 same-patient biopsies were increased *TP53*, *RB1*, and PI3K/AKT pathway alterations in mCRPC (Figure 2 and Supplemental Figure 7), suggesting that these may emerge with treatment selection pressures.

In several cases, mutations in *TP53* (*n* = 4) and *RB1* (*n* = 4) detected in mCRPC samples were not detected in the same patient’s matched, treatment-naive, and diagnostic primary tumor biopsy. Overall, there was a decrease in copy number for both *TP53* and *RB1* in mCRPC, even after adjusting for tumor purity based on low-pass WGS. More deep deletions in *PTEN* were also detected in the mCRPC cohort. Mutations in the WNT pathway genes *CTNNB1* and *APC*, as well as *MYC* amplification, were also more common in mCRPC.

Conversely, aberrations in DDR pathway genes were relatively unchanged from diagnosis to mCRPC. Eleven truncating mutations in *BRCA2*, *CDK12*, *ATM*, *MSH6*, and *PALB2* were identified in the mCRPC biopsies of 9 of 61 patients (one patient had both *CDK12* and *PALB2* mutations; one patient had *CDK12* and *MSH6* mutations). Two patients had pathogenic germline *BRCA2* mutations; in both of these cases, both the primary untreated tumor and the mCRPC biopsy presented loss of heterozygosity resulting in biallelic *BRCA2* loss. The other 9 deleterious mutations (4 in *CDK12*, 2 *BRCA2*, 1 *ATM*, 1 *PALB2*, 1 *MSH6*) were only detected in somatic DNA. All 9 of 9 were also detected in the patient-matched, metachronous, diagnostic, treatment-naive biopsies. In 3 of 4 cases with CDK12 truncating mutations, there was a second missense mutation in CDK12. Again, these second events were also detected in both the diagnostic patient-matched biopsies. However, 2 truncating mutations in ATRX and FANCM were detected only in the mCRPC samples.

With regard to copy number aberrations in DNA repair genes, we identified a trend for lower tumor suppressor gene copy number in mCRPC samples, only partially explained by the higher tumor purity of mCRPC biopsies. No deep deletions in *BRCA1/BRCA2/ATM* were identified, although changes indicating single-copy loss with disease evolution to mCRPC were detected.

Generally, the number of private events was small. An outlier case was P001, a patient with an MMR-defective prostate cancer who had the highest mutation burden, including several shared mutations between primary and mCRPC (*APC, CDK12, MSH6, ERBB4, PTEN*, and *TP53*), several private mutations only detected in mCRPC (including missense, nontruncating mutations in *APC, ATM, EZH2, JAK1*), and several private mutations of the primary tumor not detected in the later mCRPC biopsy (*CTNNB1, PRKDC*, *ERCC3*, and *ERRC6*), suggesting the presence of different clones coming from a shared origin.

## Discussion

Molecular stratification of prostate cancer promises to impact patient care and deliver more precise treatments, but several challenges remain to be addressed, including the elucidation of the genomic profiles of distinct clinical states and understanding the impact of drug resistance and tumor evolution (13, 14). Here, we show that the primary prostatic biopsies of patients who develop metastatic prostate cancer are enriched for genomic aberrations typically found in mCRPC, even before exposure to androgen deprivation. These data may help define a subset of patients with locoregional disease at diagnosis with higher risk of lethal disease; clinical trials should test if these patients may benefit from more intense therapeutic approaches. Furthermore, our data support the use of primary prostate biopsies to characterize metastatic hormone-naive prostate cancers, which may facilitate the implementation of genomic testing into clinical practice.

Defects in some DDR genes have been identified as promising predictive biomarkers for PARP inhibitors or platinum chemotherapy (15–18). The prevalence of mutations and deletions in DNA repair genes in our cohorts of patients with only locoregional disease detected at diagnosis or with metastatic hormone-naive prostate cancer was similar to what has been previously described for mCRPC. In a recent study, Marshall et al. found an increased prevalence of these mutations in higher Gleason score primary tumors, which also indirectly supports the association of these mutations with more aggressive primary tumors (19). These data in a cohort of 470 primary tumors suggest that lethal prostate cancer is enriched for DNA repair defects from diagnosis, before developing castration resistance. However, the limited number of cases with DDR gene alterations in the cohort of matched primary-metastatic biopsies, including only 4 cases with *BRCA2* mutations, prevents us from making broad conclusions with regard to the genomic evolution of these tumors. Indeed, we and others have reported subclonal homozygous deletions of DDR genes (20, 21). Detecting these subclonal deletions is technically challenging with targeted NGS assays used for patient stratification in clinical practice or in clinical trials, particularly when studying primary tumor samples with low tumor content and degraded DNA.

Alterations in TP53 were common in diagnostic biopsies in this cohort. Moreover, several loss-of-function alterations of *TP53, RB1*, and *PTEN* were detected in mCRPC biopsies but not in patient-matched, treatment-naive primary tumors. Concurrent loss of RB1 and TP53 function has been postulated to drive a phenotypic change associated with resistance to endocrine therapies (22, 23). Additionally, TP53 mutations have been associated with more aggressive disease (24–26), which may in part explain why we are observing TP53 mutations more often than expected in primary prostate cancer in this cohort of patients who all had lethal forms of the disease, even if many presented as localized tumors.

As precision medicine strategies are developed for prostate cancer patients, our findings become clinically relevant. First, our analyses indicate that *RB1* loss in the primary tumor associates with poor prognosis; these data confirm recently published results from 2 independent studies looking at genomics–clinical outcome correlations in metastatic samples (27, 28). In our series, DDR defects and particularly *BRCA2* mutations did not associate with shorter survival; however, most of these patients were enrolled into PARPi clinical trials. Data from randomized trials have confirmed the improved outcome of patients with DDR defects receiving PARPi, which needs to be taken into consideration when interpreting our results. Second, these data are critically important for designing precision medicine strategies. If DNA repair defects are already detectable in the primary tumor, there is a rationale for testing synthetic lethal strategies with PARP inhibitors or platinum, in metastatic hormone-naive prostate cancer, where the magnitude of benefit for patients could be larger. These data also support the use of diagnostic prostate cancer biopsies for patient stratification based on DNA repair gene defects in trials of men with mCRPC, as the prevalence of these alterations in primary tumors from patients with lethal prostate cancer was similar to what has been reported for metastatic disease, and in the small number of same-patient sample pairs available, DDR mutational status was concordant (29). Conversely, trials investigating novel therapeutic approaches in the TP53/RB1-deficient phenotype should take into account that a proportion of genomic aberrations in *TP53* and *RB1* are not detected when assessing diagnostic treatment-naive primary tumor specimens.

The main limitation of our study comes from having only one biopsy core available per time point and patient; we therefore could not assess spatial tumor heterogeneity. Primary prostate cancers can be multifocal, and previous studies have reported on interfoci genomic heterogeneity (30, 31). We cannot rule out that in some cases the primary tumor sample may not represent the dominant tumor clone in the primary biopsy. Hence, it is possible that some of the differences we observe in paired mCRPC biopsies may have not resulted from treatment-selective pressure but from other areas of these primary tumors. However, genomic testing in clinical practice is largely based on the analyses of single biopsy cores. With the advent of novel imaging modalities, genomic stratification of prostate cancer could be improved by better identifying aggressive areas of prostate cancer in clinical diagnostic pathways (32, 33). Another key limitation is the inability to pursue subclonality assessments using our clinically oriented targeted sequencing assay. Hence, we cannot prove if some of the gene aberrations detected in the mCRPC biopsies but not in the treatment-naive samples were already present at a subclonal level at the time of diagnosis. Regardless of whether these events emerge de novo or as a result of expansion of a subclone, the observed enrichment for certain alterations (such as *TP53* or *RB1*) in the posttreatment resistance samples supports the clinical relevance of such alterations.

In conclusion, this study describes the genomic landscape of primary prostate tumors that will evolve to lethal prostate cancer across a cohort of 470 cases, with this being characterized by higher frequencies of *TP53* and DNA repair gene aberrations. Significant differences in the detection of *AR*, *TP53*, *RB1*, and *PTEN* alterations, but not of DNA repair genes, was observed when comparing same-patient mCRPC and treatment-naive biopsies. These data are important for the genomic stratification of primary prostate cancer to identify higher risk cases, support the use of primary prostate tumor biopsies for molecular stratification of metastatic hormone-naive prostate cancer, and provide a rationale for the study of DNA repair–targeting therapies, including PARP inhibitors, in earlier stages of the disease.

## Methods

### Study design.

This analysis included all consecutive patients who gave consent between March 2015 and December 2017 for molecular characterization of prostate cancer biopsies at The Institute of Cancer Research (London, United Kingdom). These studies involved either prostate tumor samples and/or newly acquired metastatic biopsies. We report here on all patients for whom a treatment-naive primary prostate tumor sample was successfully sequenced. Primary tumor samples were retrieved from referring hospitals. In most cases, only one sample was made available for the study; if more than one sample from the primary tumor was available, the highest Gleason lesion was used. Additionally, metastatic biopsies in castrate-resistant conditions were pursued in consenting patients.

### Sample acquisition and processing.

All prostate cancer treatment-naive and metastatic biopsy samples were centrally reviewed by a pathologist. DNA was extracted from formalin-fixed and paraffin-embedded (FFPE) tumor blocks (average, 6 sections of 10 μm each per sample) using the FFPE Tissue DNA kit (Qiagen). DNA was quantified with the Quant-iT high-sensitivity PicoGreen double-stranded DNA Assay Kit (Invitrogen). The Illumina FFPE QC kit (WG-321-1001) was used for DNA quality control tests according to the manufacturer’s protocol as previously described (34). In brief, quantitative PCR (qPCR) was performed using 4 ng of sample or control DNA, and the average Cq (quantification cycle) was determined. The average Cq value for the control DNA was subtracted from the average Cq value of the samples to obtain a ΔCq. DNA samples with a ΔCq less than 4 were selected for sequencing. A double amount of DNA was used for cases with ΔCq between 2–4.

### Sequencing and bioinformatic analyses.

Libraries for next-generation targeted sequencing were constructed using a customized panel (GeneRead DNAseq Mix-n-Match Panel v2; Qiagen) covering 6025 amplicons (398702 bp) across 113 genes used in Pritchard et al. (Supplemental Table 2) (35). Libraries were run using the MiSeq Sequencer (Illumina). FASTQ files were generated using the Illumina MiSeq Reporter v2.5.1.3. Sequence alignment and mutation calling were performed using the Qiagen GeneRead Targeted Exon Enrichment Panel Data Analysis Portal (https://ngsdataanalysis.qiagen.com). Mutation calls were reviewed manually in Integrative Genomics Viewer (https://software.broadinstitute.org/software/igv) according to the standard operating procedure for somatic variant refinement of tumor sequencing data, following principles previously described (36). This manual review included assessing read strand quality, base quality, read balance, and sequencing artifacts (high discrepancy regions, adjacent indels, multiple mismatches, start or end of amplicons). Mutation annotation was based on data from publically available databases (ClinVar, COSMIC, Human Genome Mutation Database, IARC TP53 Database), published literature, and in silico prediction tools. Only deleterious mutations were included in the analysis.

Copy number variations (CNVs) in prostatic biopsies were assessed using the CNVkit (v0.3.5, https://github.com/etal/cnvkit) (37), which we previously validated in an independent cohort of prostate cancer samples (7). The read depths of tumor samples were normalized and individually compared with a reference consisting of nonmatched male germline DNA. The circular binary segmentation (CBS) algorithm was used to infer copy number segments. Quality estimation of the CNV was based on distribution of bin-level copy ratios within segments. Cases were excluded from the analysis if any of the following criteria were met: IQR greater than 1, total reads fewer than 500,000, fewer than 99.9% of reads on target, fewer than 95% paired reads, or single reads greater than 0. Manual review of copy number calls for selected oncogenes and tumor suppressors was pursued, accounting for tumor content. Oncoprints and heatmaps representing mutations and copy number calls were generated using R and cBioportal OncoPrinter (38–40).

Low-pass WGS was performed on the mCRPC, and same-patient, treatment-naive, diagnostic, paired samples for copy-number profiling. Libraries were constructed using the NEBNext Ultra FS II DNA kit (New England Biolabs) according to the manufacturer’s protocol. Samples were pooled and run on the NextSeq (Illumina) at ×0.5 mean coverage, using the 300 cycles High Output v2.5 kit (Illumina). BCL files were converted to FASTQ files using BCL2FASTQ v2.17. Sequence alignments were performed using Burrows-Wheeler Aligner (BWA-MEM v0.7.12) to the hg19 human genome build. Copy number analysis was performed using IchorCNA (41). In short, hg19 genomes (filtered centromeres) were divided into 500-kb nonoverlapping bins, and the abundance of the mapped reads was counted by HMMcopy Suite in each bin and predicted segments of CNAs. GC and mappability bias were corrected by loess regression and based on a panel of germline DNA sequencing from healthy donors. The maximum CNA detection was set to 20 copies.

Raw sequencing data have been deposited at the European Nucleotide Archive with accession number PRJEB32038. VCF files with mutation calls and CN values for the targeted sequencing data are available in the Supplemental Material.

### Statistics.

Descriptive statistics were used to summarize patient and sample characteristic data as well as mutation frequency. The prevalence of mutations was compared between cohorts using Fisher’s exact test. The statistical analysis plan and the gene list to be analyzed was designed before data collection. A Bonferroni’s correction was applied; *P* values of less than 0.01 were considered statistically significant. All tests were 2-sided unless otherwise specified.

Additionally, exploratory associations between the preselected list of gene alterations and patient outcomes were tested in a subset of the study population (*n* = 210) with available consent for clinical data collection (all at The Royal Marsden Hospital, London, United Kingdom). Clinical data were captured retrospectively from electronic patient records. Time to ADT progression was defined from the date of starting ADT to start of first mCRPC therapy. Overall survival was defined as time from the date of diagnosis, date of metastatic disease, and the date of CRPC to the date of death or last follow-up. To account for variability among patients who were diagnosed with de novo metastatic versus localized disease, survival data are presented from the first evidence of metastatic disease. Patients alive at the time of last follow-up were censored. Association of genomic aberrations with survival are presented using Kaplan-Meier curves and log-rank test. All calculations were performed usingSTATA v15.1 (Stata Corp).

### Study approval.

The study included all patients with mCRPC who, between March 2015 and December 2017, provided written consent to participate in 1 of 2 IRB-approved molecular characterization programs for prostate cancer: an internal molecular characterization study at The Royal Marsden Hospital and/or a tumor NGS prescreening study at 17 hospitals (Supplemental Material) for the TOPARP-B study, an investigator-initiated clinical trial of the PARP inhibitor olaparib in mCPRC (42) (CR-UK 11/029, NCT 01682772).

## Author contributions

JM, SC, and JSDB designed the study. JM, DD, NP, EH, and JSDB created the study methodology. JM, PR, RC, CM, S Sumanasuriya, DB, MB, AP, ZZ, MF, RPL, NT, BF, RJ, UM, CR, MV, OP, SJ, TE, and S Sandhu acquired consent from patients, acquired samples, and collected clinical data. JM, CB, IF, SM, DNR, BG, MA, and SC processed samples and generated experimental data. GS, WY, MC, and SC planned and conducted bioinformatics analysis. DD and NP designed and conducted the statistical analysis plan. JM, GS, WY, SC, NP, DD, and JSDB analyzed and interpreted data. JM, GS, SC, and JSDB wrote the manuscript. EH and JSDB obtained funding. SC and JSDB supervised the study. All authors reviewed and approved the manuscript. The order of co–first authors was determined based on their role in data interpretation and manuscript preparation.

## Supplementary Material

Supplemental data

Supplemental Data Set 1

Supplemental Data Set 2

Supplemental Data Set 3

## Figures and Tables

**Table 4:**
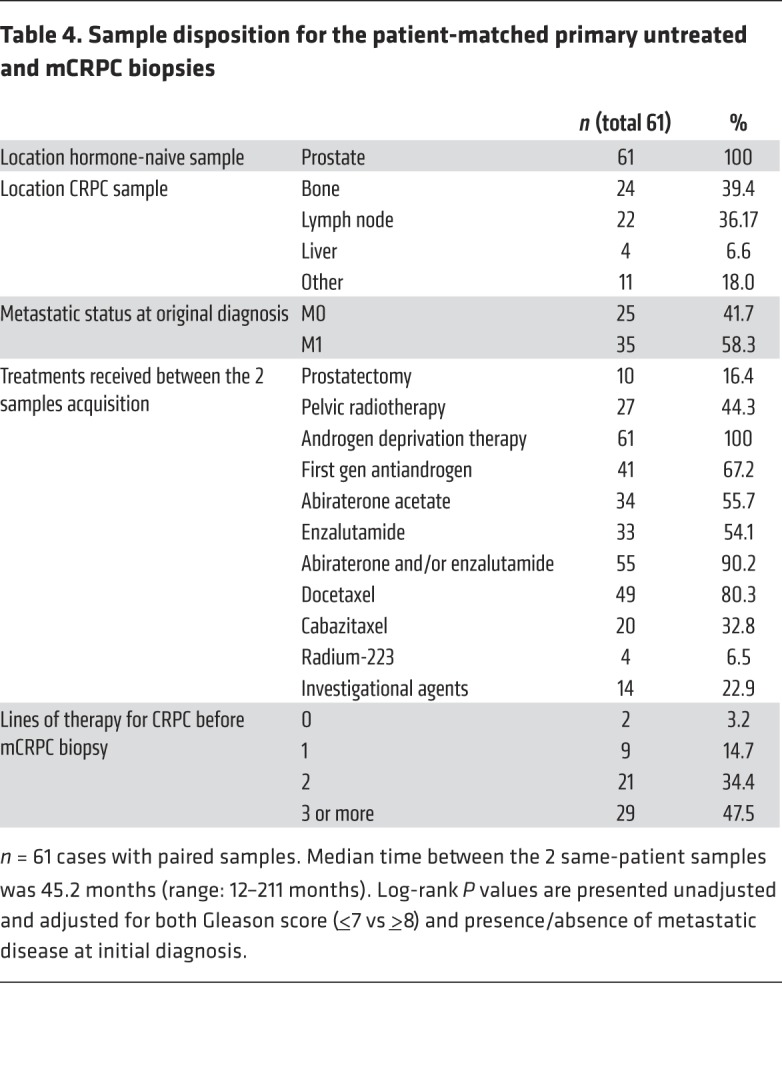
Sample disposition for the patient-matched primary untreated and mCRPC biopsies

**Table 3:**
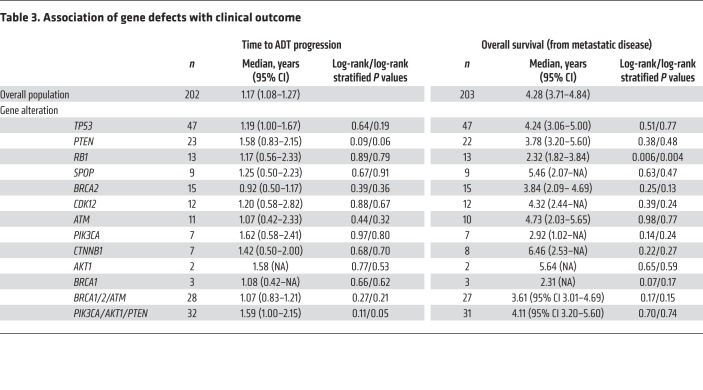
Association of gene defects with clinical outcome

**Table 2:**
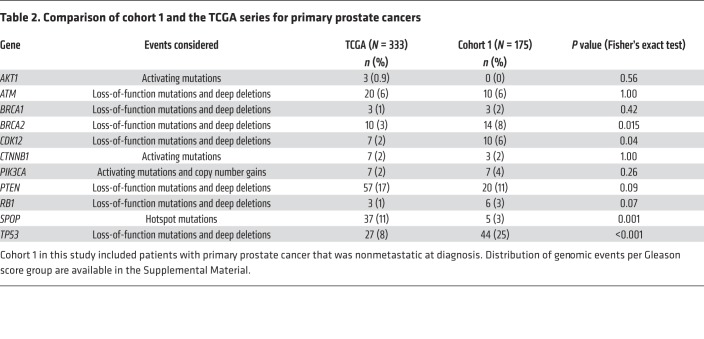
Comparison of cohort 1 and the TCGA series for primary prostate cancers

**Table 1:**
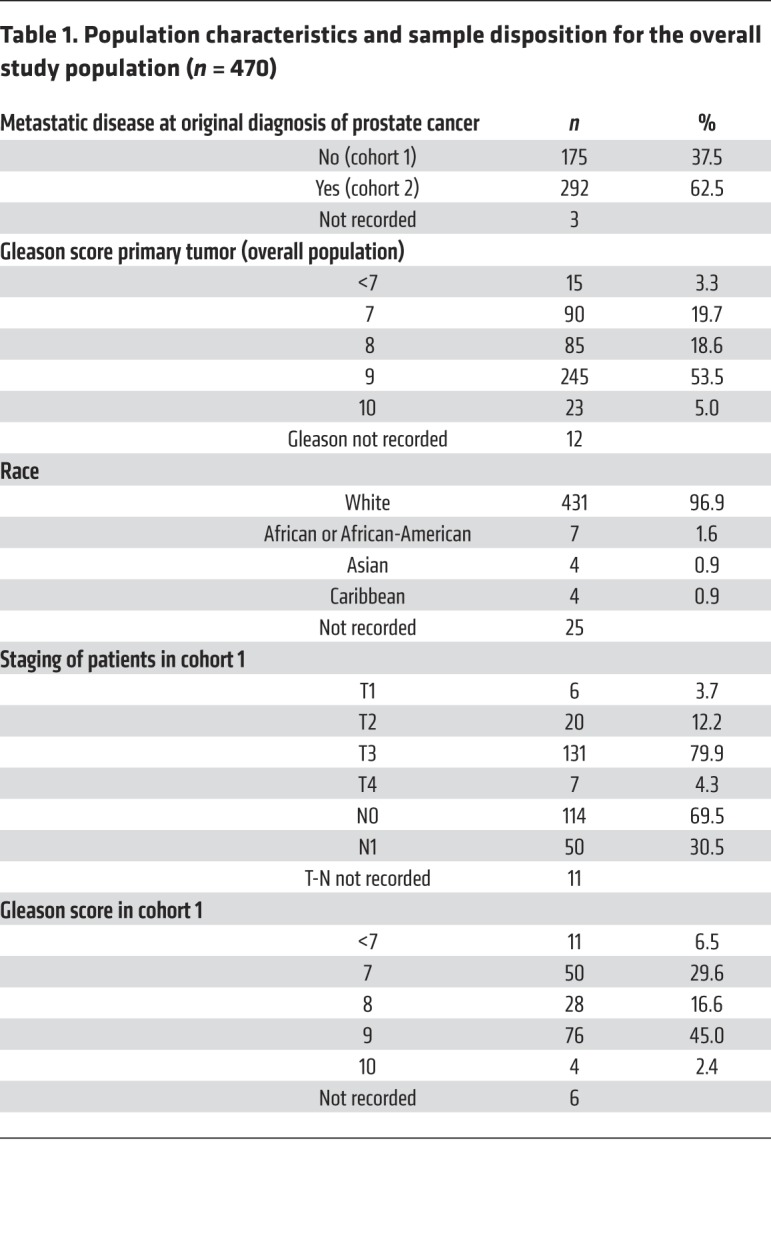
Population characteristics and sample disposition for the overall study population (*n* = 470)

## Version 1 12/24/2019

In-Press Preview

## Version 2 02/24/2020

Electronic publication

## Version 3 03/04/2020

Corrected spelling of author name, Zafeiris Zafeiriou.

## Version 4 04/01/2020

Print issue publication
