# Supplementary material for: Genomics of lethal prostate cancer at diagnosis and castration resistance
Source: J Clin Invest. 2020 Feb 24;130(4):1743–51. doi: 10.1172/JCI132031 (PMC7108902; doi:10.1172/JCI132031)

Supplementary data to

Genomics of lethal primary prostate cancer.
By Mateo J, Seed G, Bertan C et al.

Contents:

- Supplementary Tables 1 to 3
  - **Supplementary Table 1**. List of investigators and sites involved in patient consent and sample collection.
  - **Supplementary Table 2**. Areas and genes included in the customized amplicon panel
  - **Supplementary Table 3.** Comparison of the prevalence of clinically-relevant gene alterations in patients with or without synchronous metastasis at the time of original diagnosis, adjusted for Gleason score in the primary tumour.
- Supplementary Figures
  - **Supplementary Figure 1.** Consort diagram describing the sample disposition in this study.
  - **Supplementary Figure 2**. Boxplots summarizing quality control and tumor content parameters for the 470 primary tumor samples included in this cohort
  - **Supplementary Figure 3** Oncoprint for all events detected using targeted gene panel sequencing among 470 untreated primary prostate tumors from patients who later developed metastatic castration-resistant disease
  - **Supplementary Figure 4.** Overall survival curves from the time of metastatic disease for patients with and without RB1 mutations or deep deletions in the primary tumors.
  - **Supplementary Figure 5**. Violin plots representing differences in tumor purity, estimated based on low-pass WGS data, between primary and mCRPC biopsies.
  - **Supplementary Figure 6.** Individual gene violin plots for raw log2 value from low-pass WGS, indicative of copy-number changes (not adjusted)
  - **Supplementary Figure 7.** Mutation calls in the mCRPC biopsies samples and not in primary tumors from the same patients (including all genes, unsupervised clustering)
- Additional Files Submitted:
  1. VCF file with variant calls in the 470 primary prostate tumour biopsies (Cohorts 1 and 2)
  2. VCF file with log2 ratio values for copy number assessment in the 470 primary prostate tumour biopsies (Cohorts 1 and 2).
  3. VCF file with all mutation calls by targeted sequencing in patient-matched primary and mCRPC biopsies.; sample purity estimation by NGS.

Supplementary Table 1. List of investigators and sites involved in patient consent and sample collection.

**Site**	**Principal Investigator Name**
The Royal Marsden NHS Foundation Trust, Sutton	Professor Johann de Bono
University College Hospital, London	Dr Ursula McGovern
The Christie Hospital, Manchester	Dr Tony Elliott
The Beatson West of Scotland Cancer Centre, Glasgow	Professor Robert Jones
The Clatterbridge Cancer Centre, Liverpool	Dr Isabel Syndikus
St James's University Hospital, Leeds	Dr Christy Ralph
Churchill Hospital, Oxford	Professor Andrew Protheroe
Belfast City Hospital, Belfast	Dr Suneil Jain
Royal Sussex County Hospital, Brighton	Dr Angus Robinson
Royal Lancaster Infirmary, Lancaster	Dr Alison Birtle
Musgrove Park Hospital, Taunton	Dr Mohini Varughese
Royal Blackburn Hospital, Blackburn	Dr Omi Parikh
Southampton General Hospital, Southampton	Dr Simon Crabb
Airedale General Hospital, Keighley	Dr Simon Brown
Velindre Cancer Centre, Cardiff	Dr Jacob Tanguay
Western General Hospital, Edinburgh	Dr Duncan McLaren
Royal Free Hospital, London	Dr Maria Vilarino-Varela

Supplementary Table 2. Areas and genes included in the customized amplicon panel

Gene ID	Symbol	# Amplicons	Target Bases	Target Bases Covered	% Covered
207	AKT1	30	1895	1670	88%
208	AKT2	30	1706	1554	91%
238	ALK	82	5443	5050	93%
324	APC	116	9017	8950	99%
367	AR	35	3115	2490	80%
8289	ARID1A	81	7258	6132	84%
196528	ARID2	82	5928	5830	98%
472	ATM	174	10411	10282	99%
545	ATR	140	8875	8738	98%
546	ATRX	131	8249	8233	100%
8312	AXIN1	38	2997	2486	83%
8313	AXIN2	39	2732	2528	93%
580	BARD1	35	2554	2376	93%
641	BLM	64	4674	4603	98%
673	BRAF	40	2860	2456	86%
672	BRCA1	98	6118	6118	100%
675	BRCA2	152	10777	10777	100%
701	BUB1B	60	3613	3553	98%
999	CDH1	43	2969	2891	97%
51755	CDK12	67	4806	4756	99%
1019	CDK4	19	1052	1052	100%
1027	CDKN1B	9	637	626	98%
1029	CDKN2A	14	1184	713	60%
1111	CHEK1	31	1671	1671	100%
11200	CHEK2	30	2061	1892	92%
1499	CTNNB1	40	2626	2626	100%
1643	DDB2	24	1484	1484	100%
1956	EGFR	72	4564	4348	95%
4072	EPCAM	17	1125	1002	89%
2064	ERBB2	75	4356	4236	97%
2065	ERBB3	78	4720	4651	99%
2066	ERBB4	75	4552	4536	100%
2068	ERCC2	46	2816	2554	91%
2071	ERCC3	44	2649	2601	98%
2072	ERCC4	45	2971	2971	100%
2073	ERCC5	57	3861	3792	98%
2074	ERCC6	97	6684	6684	100%
2146	EZH2	46	2680	2680	100%
54855	FAM46C	14	1196	1196	100%
2175	FANCA	97	5485	5285	96%
2187	FANCB	42	2740	2740	100%
2176	FANCC	32	2152	1944	90%
2177	FANCD2	83	5351	5038	94%

2178	FANCE	27	1811	1550	86%
2188	FANCF	14	1145	1120	98%
2189	FANCG	35	2149	2144	100%
55215	FANCI	81	4811	4811	100%
55120	FANCL	29	1483	1483	100%
57697	FANCM	96	6607	6551	99%
2263	FGFR2	50	3110	3053	98%
2261	FGFR3	42	2932	2444	83%
6927	HNF1A	35	2423	2050	85%
3265	HRAS	11	780	667	86%
3716	JAK1	65	3945	3945	100%
3717	JAK2	62	3859	3854	100%
3845	KRAS	13	787	787	100%
5604	MAP2K1	26	1436	1436	100%
5605	MAP2K2	24	1423	1240	87%
6416	MAP2K4	22	1502	1263	84%
4214	MAP3K1	65	5030	4419	88%
8491	MAP4K3	71	3365	3209	95%
4193	MDM2	29	1714	1680	98%
4233	MET	69	4704	4704	100%
4292	MLH1	46	2651	2651	100%
27030	MLH3	63	4602	4602	100%
4361	MRE11A	44	2556	2556	100%
4436	MSH2	44	3277	2926	89%
4437	MSH3	63	3894	3593	92%
2956	MSH6	52	4283	3990	93%
2475	MTOR	152	9204	9204	100%
4595	MUTYH	38	1970	1962	100%
4609	MYC	19	1425	1403	98%
4615	MYD88	16	1054	1054	100%
4683	NBN	43	2585	2528	98%
4763	NF1	143	9900	9167	93%
4771	NF2	36	2164	2164	100%
4792	NFKBIA	15	1074	827	77%
4851	NOTCH1	99	8348	7005	84%
4853	NOTCH2	111	8149	7779	95%
4893	NRAS	10	650	650	100%
4914	NTRK1	45	2980	2616	88%
79728	PALB2	53	3821	3803	100%
5156	PDGFRA	60	3831	3778	99%
5290	PIK3CA	50	3607	3313	92%
5294	PIK3CG	44	3509	3296	94%
5295	PIK3R1	42	2637	2627	100%
5395	PMS2	30	2889	2126	74%
5591	PRKDC	233	14106	13829	98%
5728	PTEN	22	1392	1248	90%

10111	RAD50	65	4439	4320	97%
5890	RAD51B	30	1600	1600	100%
5889	RAD51C	24	1315	1315	100%
5892	RAD51D	22	1386	1335	96%
5925	RB1	54	3327	2965	89%
9401	RECQL4	46	4048	3191	79%
5979	RET	59	3777	3479	92%
6597	SMARCA4	88	5761	5040	87%
6598	SMARCB1	22	1392	1279	92%
8405	SPOP	22	1305	1305	100%
6714	SRC	30	1831	1555	85%
6794	STK11	23	2135	1258	59%
7128	TNFAIP3	36	2533	2533	100%
8764	TNFRSF14	19	1286	1053	82%
7157	TP53	26	1503	1396	93%
7248	TSC1	64	3915	3915	100%
7249	TSC2	103	6537	5937	91%
7428	VHL	12	702	492	70%
7486	WRN	85	4979	4976	100%
7490	WT1	24	1784	1282	72%
7507	XPA	12	942	750	80%
7508	XPC	51	3143	3015	96%
7517	XRCC3	17	1205	1047	87%
8233	ZRSR2	28	1669	1654	99%
	**Total**	2338	155986	144654	91%

Supplementary Table 3. Comparison of the prevalence of clinically-relevant gene alterations in patients with or without synchronous metastasis at the time of original diagnosis, adjusted per Gleason score in the primary tumour.

Gene alteration	Overall		Per cohort				Per Gleason				Cohort 1 per Gleason				Cohort 2 per Gleason			
			Cohort 1		Cohort 2		<=7		>=8		<=7		>=8		<=7		>=8	
Total	*470*	*100,0%*	*175*	*100,0%*	*292*	*100,0%*	*105*	*100,0%*	*353*	*100,0%*	*61*	*100,0%*	*108*	*100,0%*	*44*	*100,0%*	*243*	*100,0%*
AKT1	5	1,1%	0	0,0%	4	1,4%	1	1,0%	4	1,1%	0	0,0%	0	0,0%	1	2,3%	3	1,2%
ATM	18	3,8%	10	5,7%	8	2,7%	5	4,8%	13	3,7%	5	8,2%	5	4,6%	0	0,0%	8	3,3%
BRCA1	5	1,1%	3	1,7%	2	0,7%	0	0,0%	5	1,4%	0	0,0%	3	2,8%	0	0,0%	2	0,8%
BRCA2	31	6,6%	14	8,0%	17	5,8%	9	8,6%	22	6,2%	6	9,8%	8	7,4%	3	6,8%	14	5,8%
CDK12	22	4,7%	10	5,7%	12	4,1%	**1**	**1,0%**	**21**	**5,9%**	1	1,6%	9	8,3%	0	0,0%	12	4,9%
CTNNB1	15	3,2%	3	1,7%	12	4,1%	5	4,8%	10	2,8%	2	3,3%	1	0,9%	3	6,8%	9	3,7%
PIK3CA	18	3,8%	7	4,0%	11	3,8%	5	4,8%	13	3,7%	3	4,9%	4	3,7%	2	4,5%	9	3,7%
PTEN	56	11,9%	20	11,4%	36	12,3%	10	9,5%	44	12,5%	3	4,9%	15	13,9%	7	15,9%	29	11,9%
RB1	23	4,9%	5	2,9%	18	6,2%	4	3,8%	19	5,4%	2	3,3%	3	2,8%	2	4,5%	16	6,6%
SPOP	33	7,0%	**5**	**2,9%**	**28**	**9,6%**	7	6,7%	26	7,4%	1	1,6%	4	3,7%	6	13,6%	22	9,1%
TP53	127	27,0%	44	25,1%	82	28,1%	21	20,0%	105	29,7%	15	24,6%	29	26,9%	6	13,6%	76	31,3%

In bold: significant comparisons at the 0.05 level, Fisher exact test (M0 vs M1, Gleason <=7 vs >=8 separately

Supplementary Figure 1. Consort diagram describing the sample disposition in this study.

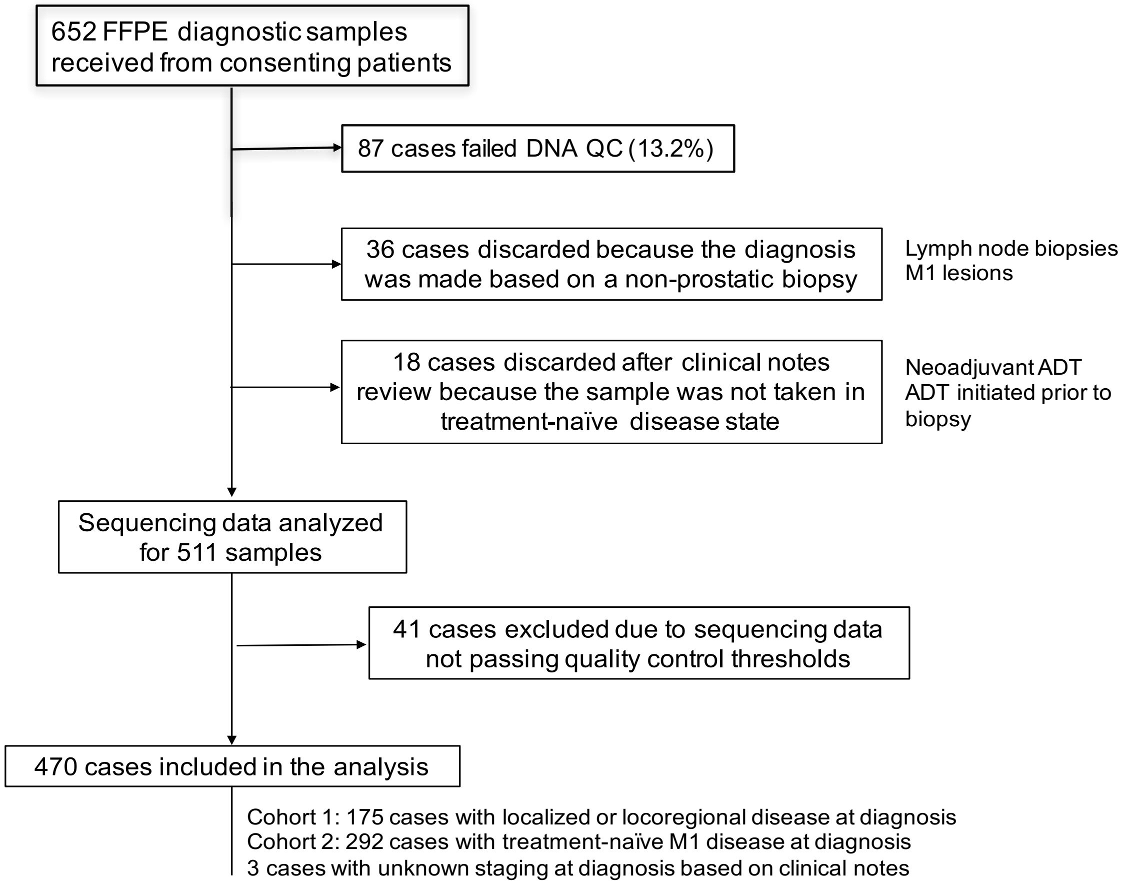

Supplementary Figure 2. Boxplots summarizing quality control and tumor content parameters for the 470 primary tumor samples included in this cohort.

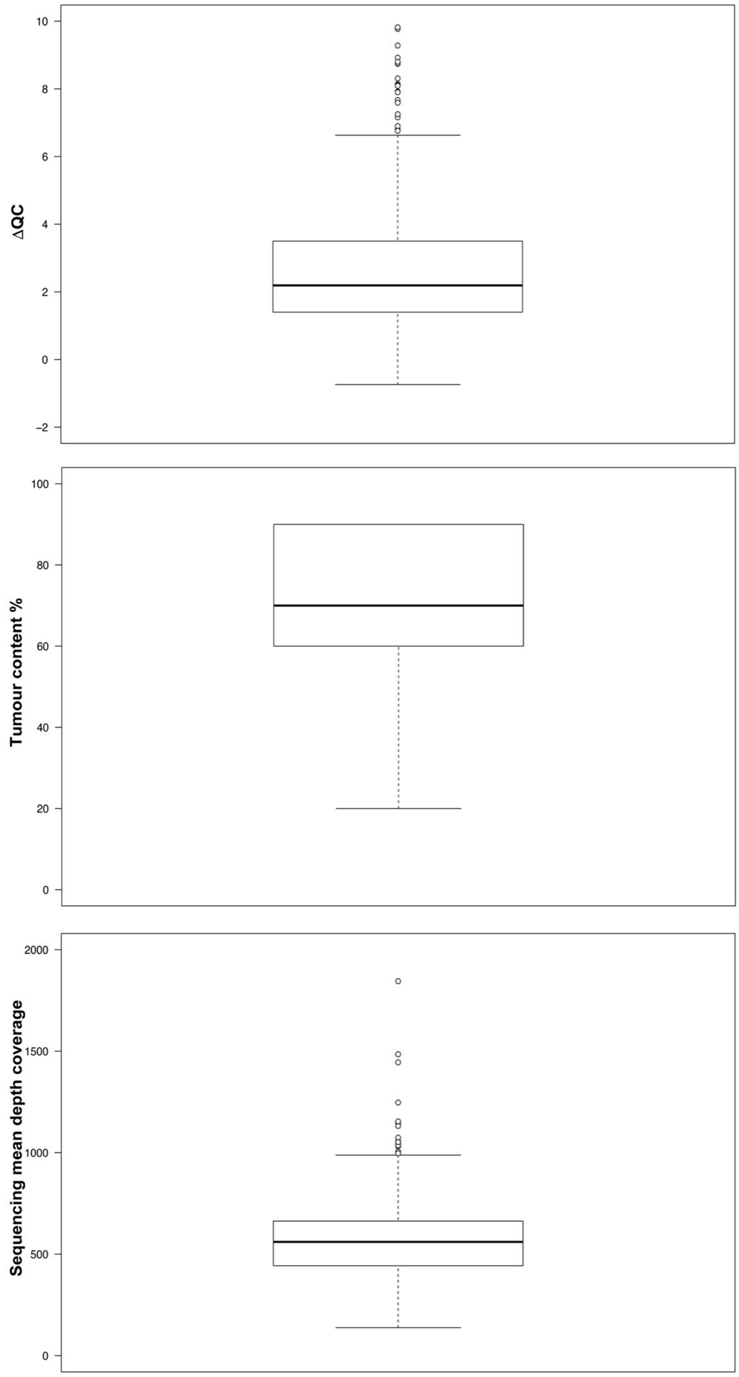

Supplementary Figure 3. Oncoprint for all events detected using targeted gene panel sequencing among 470 untreated primary prostate tumors from patients who later developed metastatic castration-resistant disease.

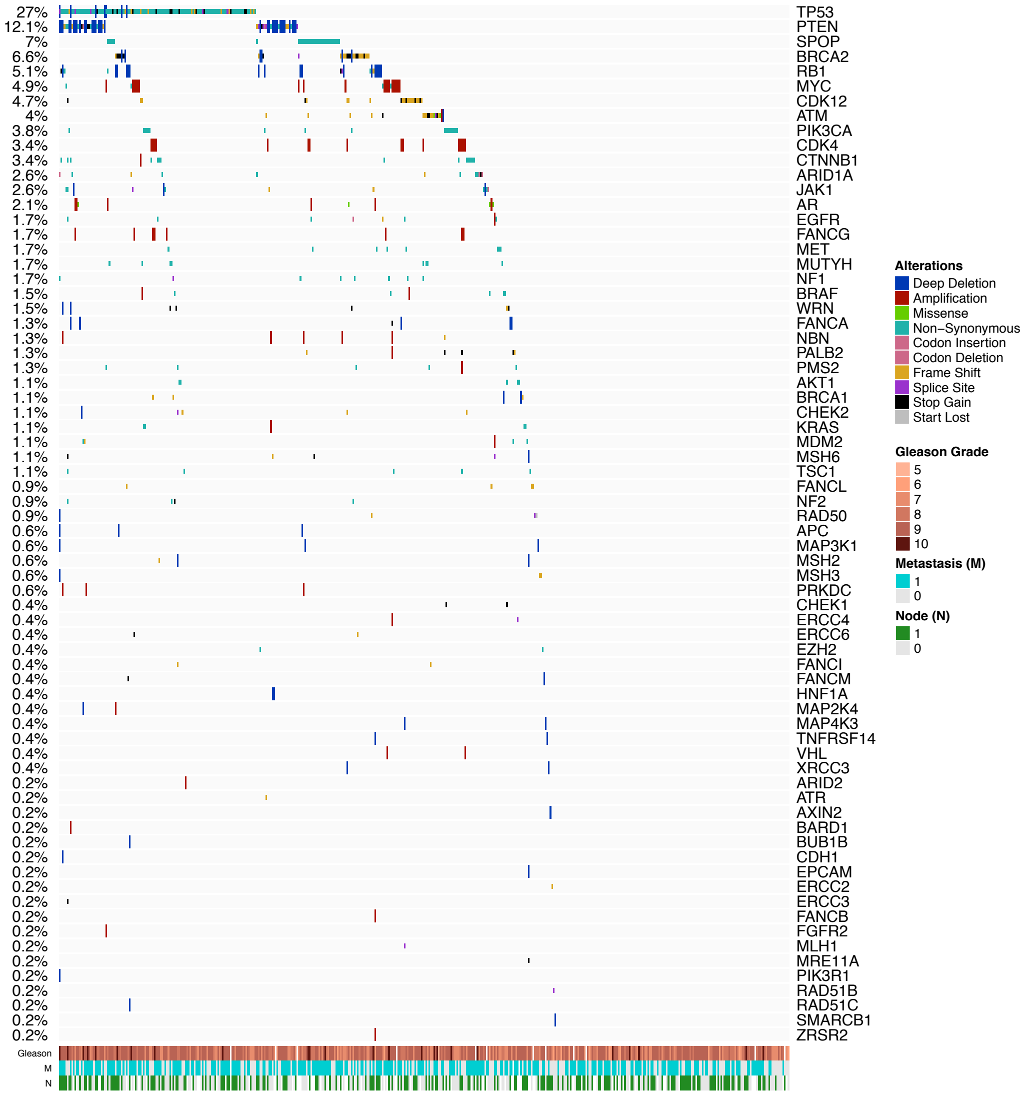

Supplementary Figure 4. Overall survival curves from the time of metastatic disease for patients with and without RB1 mutations or deep deletions in the primary tumors.

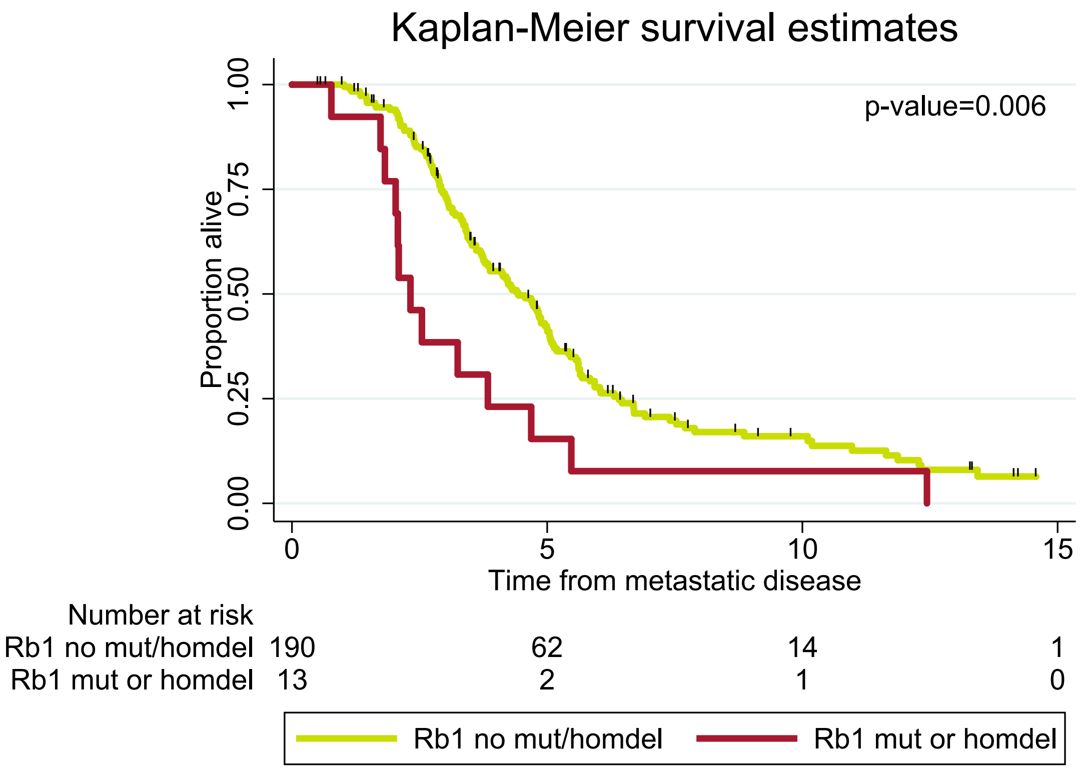

Supplementary Figure 5. Violin plots representing differences in tumor purity, estimated based on low-pass WGS data, between primary and mCRPC biopsies.

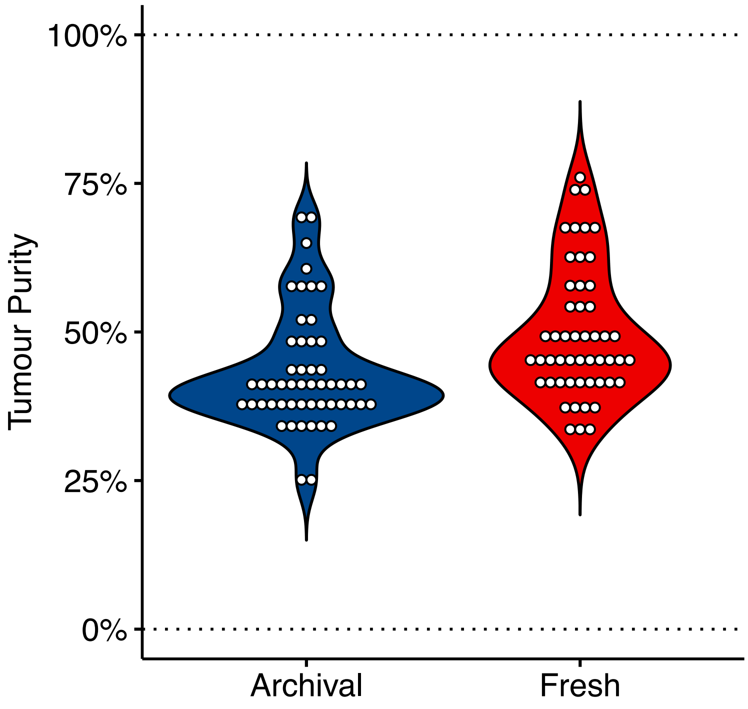

Supplementary Figure 6. Individual gene violin plots for raw log2 value from low-pass WGS, indicative of copy-number changes (not adjusted)

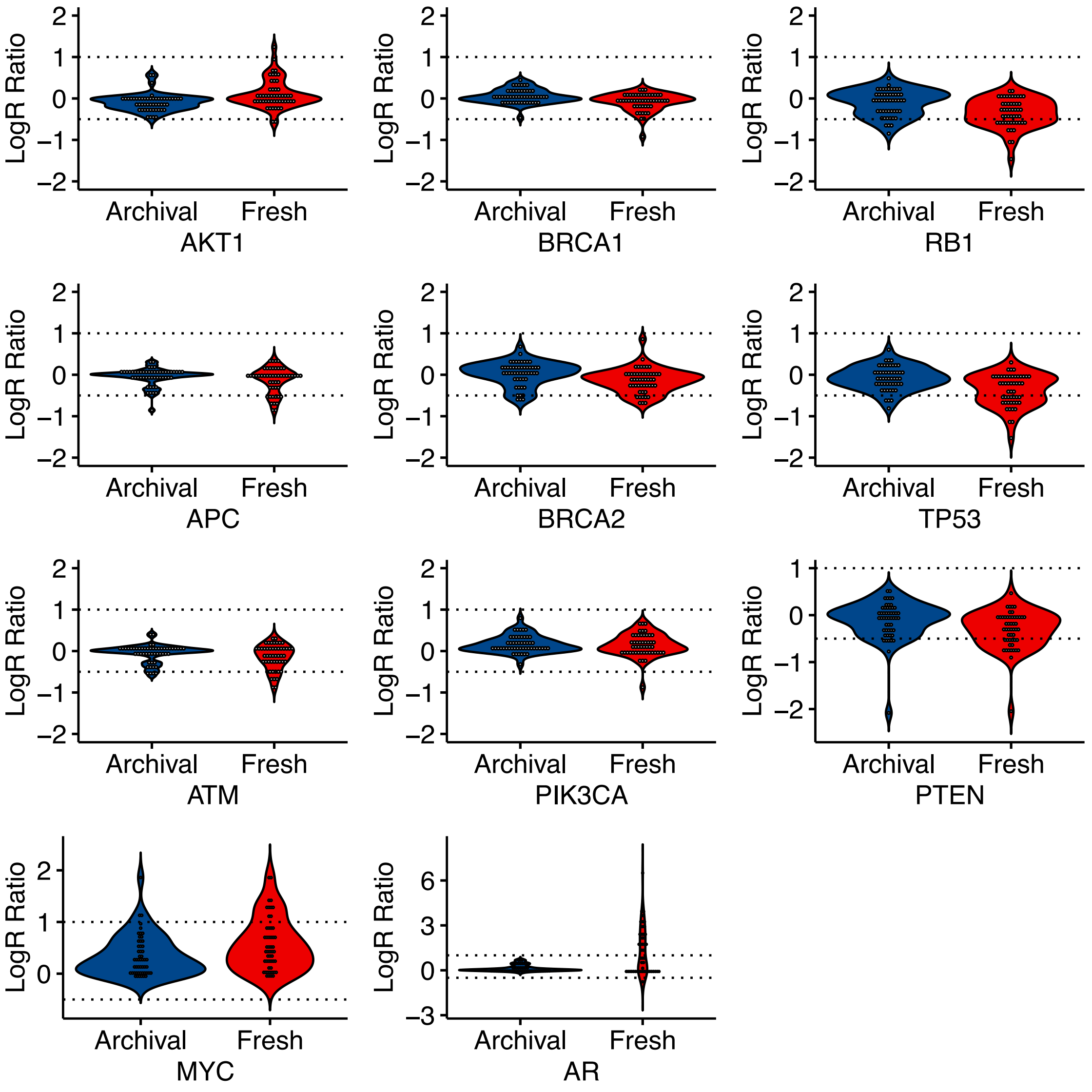

Supplementary Figure 7. Mutation calls in the mCRPC biopsies samples and not in primary tumours from the same patients (including all genes)

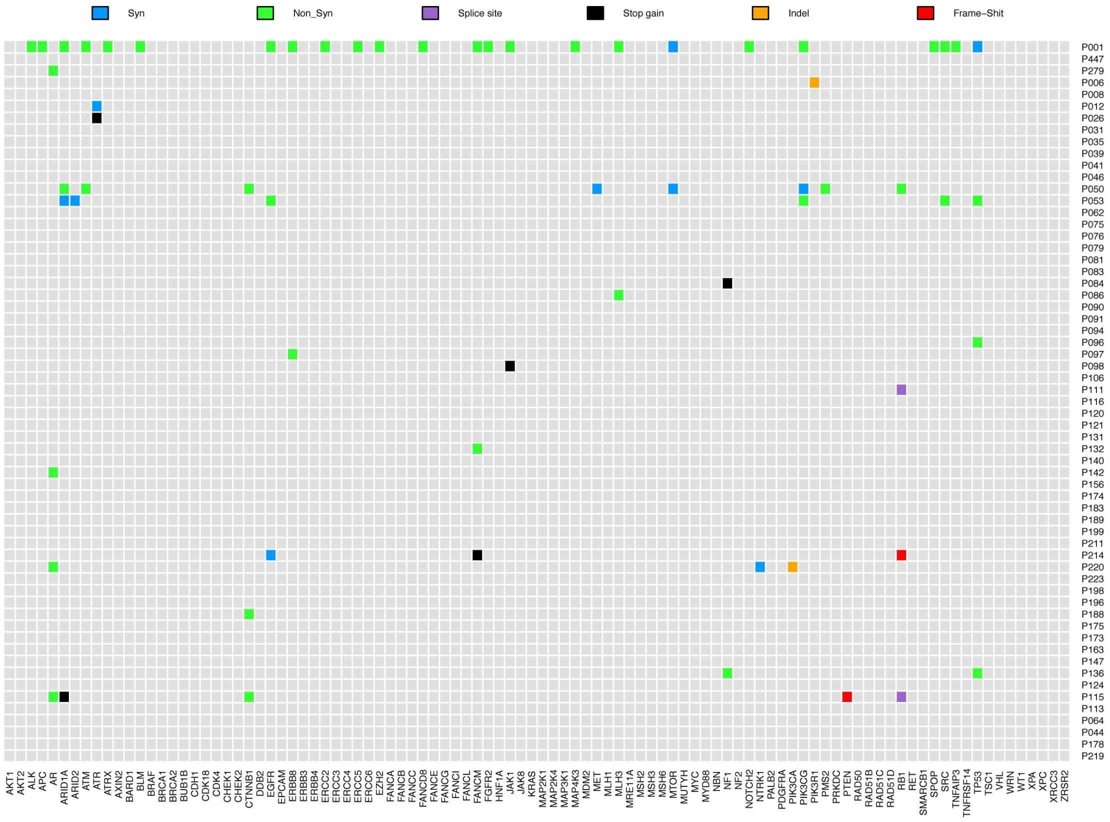

Supplement: Supplemental data [file jci-130-132031-s099.pdf]
